# Institutional Notes and News

**Published:** 1909-10-09

**Authors:** 


### Institutional Notes and News.

### NATIONAL HOSPITAL, QUEEN SQUARE.

Lord Mount Edgcumbe writes to the *Plymouth Mercury* calling attention to the Jubilee Fund for the National Hospital for the Paralysed and Epileptic, Queen Square. In the course of his letter he remarks as follows: "The chief part of the hospital is a memorial to the late Duke of Albany. The object of the Jubilee Fund is an extension of the building and its usefulness in studying the best methods of treating all forms of nervous diseases, which are so prevalent, and cause so much life-long misery. The sum for which an appeal is being made throughout the country is £50,000. No deduction whatever for expenses will be allowed from any gifts to this Jubilee Fund. The annual number of in-patients has been about 1,000, and out-patients 7,000. I find that seventy-three patients actually coming from Cornwall have been in-patients of the hospital, and no doubt if we knew the number of Cornish people not resident in the county that have benefited by it, either as in-patients or out-patients, the number would be very considerable." On October 9 the Princess of Wales will attend the hospital and receive purses. On November 4 his Majesty opens the new wing.

### GEMESLEY INFECTIOUS HOSPITAL.

The Glossop Town Council has decided not to go forward with a proposal to spend more money on altering and extending the borough infectious hospital at Gemesley. Dr. Mackenzie (the Medical Officer of Health) said that the hospital had been a real benefit in dealing with epidemics. The statements which medical gentlemen had made that the building was so impregnated with germs that it could only be disinfected by being burnt down were not proved by facts.

### UNIVERSITY COLLEGE HOSPITAL'S PRIZE-GIVING.

At the opening of the session and presentation of prizes at University College Hospital, Mr. C. E. Shattock, one of the hospital students, obtained nine successes. These included the Atchison scholarship, the Bruce medal for proficiency in surgery and pathology, and seven prizes for class examinations in various medical subjects. Mr. Shattock is the son of Mr. S. G. Shattock, the pathological curator of the Royal College of Surgeons and St. Thomas's Hospital Museums.

### THE KING AND THE ROYAL CHELSEA HOSPITAL.

The King has been pleased, by Warrant dated September 9th, to cancel the Warrant dated December 19th, 1898, regulating the government of the Royal Hospital, Chelsea, and to promulgate revised regulations relating to the establishment, duties, salaries, allowances, and other matters affecting the officers and others employed at that hospital.

### NORTH EVINGTON INFIRMARY.

The Leicester Guardians have called upon the matron of this infirmary to resign. She has contented herself with acknowledging the receipt of the communication. This is now supplemented by a largely-signed petition from the staff, asking the Board to reconsider its decision, and paying a high tribute to the matron's characteristics. The Board, at its last meeting, decided to allow the memorial to lie on the table. But it was also agreed that in the event of a vacancy the Local Government Board should be invited to amend the order under which the infirmary is managed. What the outcome of the present embarrassing difficulty will be has yet to be seen. About one thing everybody is agreed. It has become imperative to make a radical change in the staff, with the object of eliminating all friction, and insuring not only the efficient, but smooth, working of the establishment.

### MATER INFIRMORUM HOSPITAL, BELFAST.

A new nursing home in connection with this hospital has been opened in Crumlin Road, facing the hospital. The premises have been acquired at considerable cost and have undergone a complete transformation, the alterations and additions being on a most extensive scale. The ground floor is occupied principally by the sitting, reception, and dining-rooms, all of which are large and airy; while at the other end there is a well-equipped kitchen, pantry, and scullery. A recreation-room is also provided. The first, second, and third floors are occupied by the bedrooms, and on each landing there are up-to-date and admirably equipped bathrooms. The building accommodates between 30 and 40 nurses, has a frontage of about 70 feet, and is three and a half stories high. The material used in the building is perforated brick. The contract for the building was given to Messrs. F. Savage and Sons, Lancaster Street, who carried out their work in a most satisfactory manner. The entire work was carried out according to the plans prepared by Messrs. Moore and Flanigan, architects, Royal Avenue.

### SOUTHPORT HOMŒOPATHIC DISPENSARY AND COTTAGE HOSPITAL.

The attendances at the dispensary from January 1 to September 11 of this year exceeded 3,350, an increase over the total attendance for the preceding year. The Southport Cottage Hospital is approaching its completion, and the committee hopes to see it ready for occupation by the end of the year. The building is in every way satisfactory, and the committee has no hesitation in appealing to all who are interested in homœopathy, and to the much larger public to whom the health of the community is of supreme importance, to contribute as liberally as possible to the building fund, so that the debt of nearly £3,000 may be wiped out before the hospital is opened.

---

At St. George's Hospital Medical School the University Entrance Scholarships of 70 guineas and £50 respectively have been added together and divided equally between Mr. E. F. W. Grellier, Downing College, Cambridge, and Mr. W. E. Waller, University College, Oxford.

The following entrance scholarships have been awarded at London Hospital Medical College: Price Entrance Scholarship in Science, £120, Mr. J. Bostock; Entrance Science Scholarships, £60, Mr. A. G. Winter, £35, Mr. R. J. M. Love; Epsom Scholarship, £126, Mr. H. G. Winter; Price Scholarship in Anatomy and Physiology (University Scholarship), £60, Mr. J. R. Marrack, St. John's College, Cambridge.

At the Middlesex Hospital Medical School the following entrance scholarships have been awarded:—(1) A. M. Wheeler, (2) T. C. Kidner, (3) A. O. Gray; University, T. L. Hardy and H. J. Shields (equal), Freer Lucas (Epsom College scholar), C. E. Procter; New Zealand, T. Harrison.

At Guy's Hospital Medical School the following entrance scholarships and certificates have been awarded:—Senior Science Scholarship for University students, £50, Trevor Braby Heaton, B.A., Christ Church Oxford. Junior Science Scholarships, £150, Charles Hamilton Gould, Preliminary Science Class, Guy's Hospital, and B.H.S., Christchurch, New Zealand; £60, James York Moore, Preliminary Science Class, Guy's Hospital. Equal certificates, Allan Noel Minns, Thetford Grammar School, and Cyril Henry Edwards, St. Paul's School, West Kensington. Entrance Scholarships in Arts, £100, Henry Francis Thomas Hogben, Bedford Grammar School, and William Morris Lansdale, St. Olave's and St. Saviour's Grammar School (equal); £50, James Kyle, University Tutorial College.

The London Inter-Collegiate Scholarships Board announce awards of scholarships and exhibitions: King's College, Sambrooke Medical Exhibition, £100, C. F. Hacker, University College, London. Westminster Hospital Medical School, "Guthrie" Art Scholarship, £60, M. C. Breese, Dulwich College; Natural Science Scholarship, £60, H. J. Hoyte, University College, Nottingham. London (Royal Free Hospital) School of Medicine for Women, St. Dunstan's Medical Exhibition, £180, Miss North Hamill, St. Paul's Girls' School; one scholarship, £30, Miss Lyndall Rice, formerly of Girls' High School, Brisbane.

Arrangements have been made by the Society for the Study of Inebriety for the following meetings to be held in the rooms of the Medical Society of London, 11 Chandos Street, Cavendish Square, W.: Tuesday, October 12, 1909 (afternoon meeting), J. Milne Bramwell, M.D., C.M. author of "Hypnotism: Its History, Practice and Theory," will open a discussion on "Suggestion, and its Rôle in the Treatment of Inebriety." Tuesday, January 11, 1910 (afternoon meeting), Albert Wilson, M.D., author of "Education, Personality, and Crime," will open a discussion on "Alcoholism and Crime." Tuesday, April 12, 1910 (afternoon meeting), William Bingham, Esq., J.P., managing director of "The Sceptre Life Association," will open a discussion on "Alcohol and Life Assurance."

